# Effects of Xiaoyaosan on Stress-Induced Anxiety-Like Behavior in Rats: Involvement of CRF1 Receptor

**DOI:** 10.1155/2016/1238426

**Published:** 2016-03-02

**Authors:** You-Ming Jiang, Xiao-Juan Li, Zhen-Zhi Meng, Yue-Yun Liu, Hong-Bo Zhao, Na Li, Zhi-Yi Yan, Qing-Yu Ma, Han-Ting Zhang, Jia-Xu Chen

**Affiliations:** ^1^School of Preclinical Medicine, Beijing University of Chinese Medicine, No. 11, Beisanhuan Donglu, Chaoyang, Beijing 100029, China; ^2^Department of Behavioral Medicine & Psychiatry, West Virginia University Health Sciences Center, Morgantown, WV 26506-9137, USA

## Abstract

*Background*. Compared with antidepressant activity of Xiaoyaosan, the role of Xiaoyaosan in anxiety has been poorly studied.* Objective*. To observe the effects of Xiaoyaosan on anxiety-like behavior induced by chronic immobilization stress (CIS) and further explore whether these effects were related to CRF1R signaling.* Methods*. Adult male SD rats were randomly assigned to five groups (*n* = 12): the nonstressed control group, vehicle-treated (saline, p.o.) group, Xiaoyaosan-treated (3.854 g/kg, p.o.) group, vehicle-treated (surgery) group, and antalarmin-treated (surgery) group. Artificial cerebrospinal fluid (0.5 *μ*L/side) or CRF1R antagonist antalarmin (125 ng/0.5 *μ*L, 0.5 *μ*L/side) was bilaterally administered into the basolateral amygdala in the surgery groups. Except for the nonstressed control group, the other four groups were exposed to CIS (14 days, 3 h/day) 30 minutes after treatment. On days 15 and 16, all animals were subjected to the elevated plus-maze (EPM) and novelty suppressed feeding (NSF) test. We then examined the expression of CRF1R, pCREB, and BDNF in the amygdala.* Results*. Chronic pretreatment with Xiaoyaosan or antalarmin significantly reversed elevated anxiety-like behavior and the upregulated level of CRF1R and BDNF in the amygdala of stressed rats. pCREB did not differ significantly among the groups.* Conclusions*. These results suggest that Xiaoyaosan exerts anxiolytic-like effects in behavioral tests and the effects may be related to CRF1R signaling in the amygdala.

## 1. Introduction

The prescription of Xiaoyaosan was originally recorded in* Taiping Huimin Hejiju Fang, *a book that was compiled in the Song Dynasty (960–1127 A.D.) by the National Medical Ministry. The book shows that Xiaoyaosan promotes the flow of liver Qi and cures “Yu-syndrome” characteristic of melancholy and depression [1, 2]. In China, the herbal remedy has been widely prescribed for hysteria, mental stress, and manic-depressive disorder [3]. Animal studies revealed that Xiaoyaosan exerted antidepressive-like effects in tail suspension and forced swimming tests [4, 5]. Compared with the antidepressant activity of Xiaoyaosan, the role of Xiaoyaosan in anxiety has been poorly studied. Our preliminary study found that chronic pretreatment with Xiaoyaosan produced an anxiolytic-like effect on behavior in rats after exposure to chronic immobilization stress (CIS) [6]. Based on our previous research, in the current study, we assessed the anxiolytic-like effects of chronically administered Xiaoyaosan on rats after exposure to CIS.

As a major hormone and critical neurotransmitter in the hypothalamic pituitary adrenocortical (HPA) axis, corticotropin-releasing factor (CRF) is considered the main neuropeptide in stress regulation [7, 8]. It has been hypothesized that CRF-1 receptor (CRF1R), which is widely expressed in the pituitary and brain, may be intimately involved in anxiety behavior [9]. Several signs of elevated anxiety were found after CRF was centrally administered or in transgenic mice overexpressing CRF [10]. Conversely, centrally administered antagonist of CRF receptor exerted anxiolytic effects in rats [11, 12]. These data indicate that enhanced anxiety-like behaviors occur upon activation of CRF1R.

Although it has been demonstrated that the levels of CRF1R mRNA transcripts were elevated in the hypothalamic paraventricular nucleus (PVN) and amygdala after exposure to immobilization stress in rats [13, 14], little is known about the effect of Xiaoyaosan on the expression of CRF1R in rats after exposure to immobilization stress. The critical function of CRF1R in anxiety behavior and anxiolytic-like effect of Xiaoyaosan raise the question whether CRF1R contributes to the regulation of Xiaoyaosan on anxiety-like behavior in rats after exposure to CIS. Thus, in the present study, we examined CIS-induced anxiety-like behavior in rats using the elevated plus-maze (EPM) and novelty suppressed feeding (NSF) test and further examined CRF1R expression in the amygdala and the role of the CRF1R signaling pathway in enhanced anxiety-like behavior.

## 2. Materials and Methods

### 2.1. Animals

Healthy adult male Sprague-Dawley (SD) rats (Harlan, Indianapolis, IN), initially weighing between 230 g and 250 g, were used in the study. Rats were housed in a temperature-controlled (24 ± 1°C) and light-controlled (lights on 6:00 Am–6:00 Pm) environment. Two rats were assigned to each cage and the rats had access to food and water* ad libitum*. The animals were allowed 7 days to accommodate to the environment and were brought to the experiment room 30 min prior to each experiment. All behavioral experiments were carried out between 9:00 Am and 5:00 Pm. Housing and behavioral procedures were approved by the Animal Care and Use Committee of West Virginia University Health Sciences Center and conformed to the “NIH Guide for the Care and Use of Laboratory Animals” [15].

### 2.2. Surgical Techniques

A drug infusion cannula was implanted above the basolateral amygdala (BLA) in 24 rats by stereotaxic surgery. After intraperitoneal anesthesia with ketamine (100 mg/kg) and xylazine (6 mg/kg), the rats were placed in the stereotaxic apparatus (Stoelting, Wood Dale, IL) on a heating pad. The incisor bar was set at −3.3 mm to make the skull flat. Under aseptic conditions, a 23 gauge guide cannula (Plastics One, Roanoke, VA) was inserted into the BLA bilaterally (2.9 mm posterior to the bregma, 5.0 mm lateral to the midline, and 8.5 mm ventral to the skull surface) [16]. Two screws were then placed into the skull as anchors. The guide cannula was secured using dental acrylic. After surgery, ketoprofen (10 mg/kg, im) was administered. Three days were allowed for rats to recover prior to subsequent experimental procedures.

### 2.3. Drugs and Drug Administration

Xiaoyaosan contains eight herbs including* Poria cocos* (Schw) Wolf (Poria) 300 g,* Paeonia lactiflora *Pall (*Radix paeoniae alba*) 300 g,* Glycyrrhiza uralensis *Fisch (*Radix glycyrrhizae*) 150 g,* Bupleurum chinense DC* (*Radix bupleuri*) 300 g,* Angelica sinensis* (Oliv.)* Diels* (*Radix angelicae sinensis*) 300 g,* Atractylodes macrocephala *Koidz. (*Rhizoma atractylodis macrocephalae*) 300 g,* Mentha haplocalyx *Briq. (*Herba menthae*) 100 g, and* Zingiber officinale *Rosc. (*Rhizoma zingiberis ecens*) 100 g. The herbs were authenticated by Dr. B. Liu, Department of Botany, Beijing University of Chinese Medicine, according to the size, shape, texture, color, and odor of the samples specified in the* Pharmacopoeia of the People's Republic of China 2010 Edition, Volume I* before purchase from Tongrentang Drugstore in Beijing. Xiaoyaosan was extracted and processed at Sino-Japan Friendship Hospital (Beijing). The extraction procedures of Xiaoyaosan were described previously [17–19]. For oral administration (via gavage, p.o.), Xiaoyaosan was dissolved in saline and diluted to the desired concentration. Xiaoyaosan (3.854 g/kg, p.o.) was administered daily for 14 days. The dose was found to be effective from our previous studies [17–19].

CRF1R antagonist antalarmin (Tocris Bioscience, Ellisville, MO) was dissolved in artificial cerebrospinal fluid (aCSF) (containing 35% acetonitrile and 0.1% trifluoroacetic acid) at the final concentration of 125 ng/0.5 *μ*L [20–22] and injected into the BLA using 30-gauge microinjection cannulas (Plastic One), which can fit into the guide cannulas and extended 1 mm beyond. Antalarmin or aCSF (as vehicle) was administered bilaterally at a rate of 0.5 *μ*L/min by using a microinfusion pump. The cannula was left in place for additional 60 seconds after microinfusion to allow diffusion. The procedure was performed daily for 14 days.

### 2.4. Chronic Immobilization Stress (CIS)

Sixty rats were randomly assigned to five groups (*n* = 12 per group) as follows: the nonstressed control group, the vehicle-treated (saline, p.o.) group, the Xiaoyaosan-treated (3.854 g/kg, p.o.) group, the vehicle-treated (surgery) group, and the antalarmin-treated (surgery) group. CIS was performed 30 min after drug administration by using a soft rodent restrainer made of plastics that closely fit to the rats' body with two adjustable soft belts, respectively, fixing the chest and abdomen. During CIS, rats were kept in the prone position for 3 h every day, between 9:00 AM and 5:00 PM, for 14 consecutive days. On day 15-16, rats were subjected to the EPM and NSF test. The rats were sacrificed between 2:00 PM and 5:00 PM after behavioral experiments to minimize diurnal fluctuation [20].

### 2.5. Elevated Plus-Maze (EPM) Test

The EPM apparatus consisted of several parts including one central part (8 × 8 cm), two opposing open and closed arms (30 × 8 cm), and nontransparent walls (30 cm in height). The experiment room was light and temperature-controlled. Between every session, both the platform and the wall were thoroughly cleaned using 10% ethanol spray. Rats were placed individually in the center of the maze (50 cm above the floor) facing an open arm. The EPM test was videotaped and recorded by using a video camera. Indices including total distance moved, number of entries into the open and closed arms, and total time spent in the open and closed arms were calculated and analyzed using the ethovision software (Noldus). Percentage of time spent on and number of entries to open arms were examined as the standard anxiety indices. Total distance moved was detected as the indicator of locomotor activity.

### 2.6. Novelty Suppressed Feeding (NSF) Test

The NSF test was performed as described previously with minor modification [23]. The NSF apparatus was a white, plastic, open chamber (50*∗*50*∗*40 cm), with 2 cm wooden bedding placed on the floor. Four pellets of food (regular chow) were placed in the center. After food deprivation for 24 h, rats were individually placed in the corner of the white chamber. The latency to begin to chew the pellets was recorded for up to 5 min.

### 2.7. Western Blotting Assays

The protein levels of CRFR1, pCREB, and BDNF in the amygdala were detected of four rats from five groups each. After behavior experiments, the brain tissue was dissected and stored at −80°C until analysis. The procedures for processing the brain tissue and Western blotting analysis were both conducted as described previously [24, 25]. Denatured samples were electrophoresed by using the sodium dodecyl sulfate-polyacrylamide (SDS-PAGE) gel, and then transferred to a PVDF membrane by using an electroblotting apparatus (Bio-Rad, Hercules, CA, USA). After nonspecific blocking, the membrane was incubated overnight at 4°C with a primary antibody (mouse anti-*β*-actin monoclonal antibody diluted to 1 : 1000, or goat anti-CRF1R polyclonal antibody diluted to 1 : 500). After wash, the membranes were incubated with secondary antibody (goat anti-mouse antibody diluted to 1 : 10000) for 1 h. Protein bands were examined at 800 nm wavelength by using a fluorescence scanner of Odyssey Infrared Imaging System.

### 2.8. Statistical Analysis

Data were expressed as mean ± SEM and analyzed using one-way analysis of variance (ANOVA) followed by Bonferroni's multiple comparison tests. The level of significance for all statistical tests was set to 0.05.

## 3. Results

### 3.1. Effect of Xiaoyaosan on Rat Performance in the EPM Test

**Figure 1:**
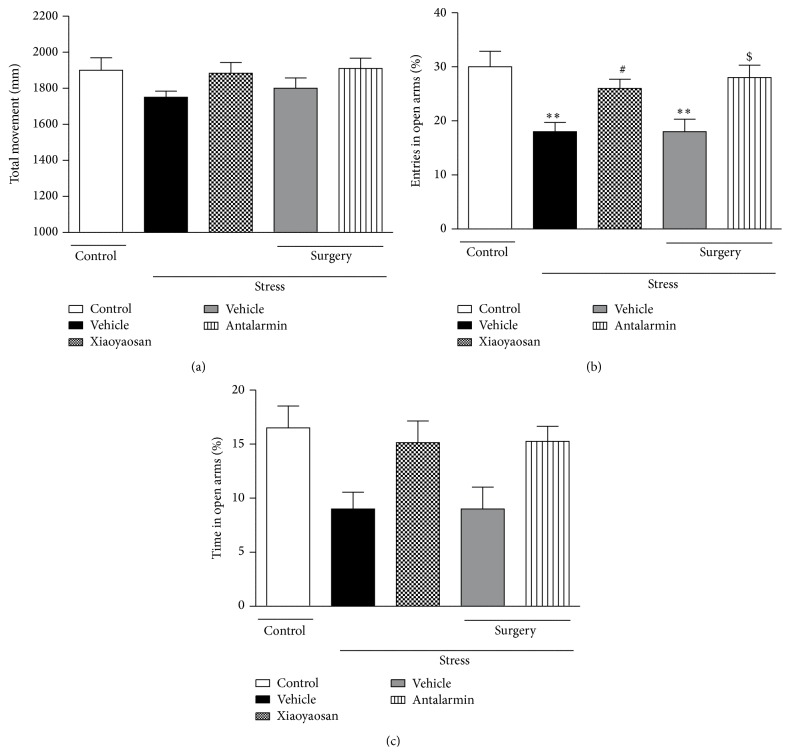
Elevated plus maze test. (a) The total distance moved. (b) The percentage of entries in open arms. (c) The percentage of time spent in the open arms. Values are mean ± SEM with 10 rats in each group. ^*∗∗*^ *p* < 0.01 versus the nonstressed control group. ^#^ *p* < 0.05 versus the vehicle-treated CIS group. ^$^ *p* < 0.05 versus the vehicle-treated CIS group after surgery.

The role of Xiaoyaosan in the regulation of anxiety-like behaviors was assessed using the EPM test. For the percentage of entries in open arms (Figure 1(b)), the stressed groups treated with vehicle (saline, p.o. or aCSF, intra-BLA infusion) showed enhanced anxiety-like behavior as indicated by decrease in the percentage of entries in open arms compared with the nonstressed control group (*p* < 0.01). Rats treated with Xiaoyaosan (3.854 g/kg, p.o.) or antalarmin (125 ng/0.5 *μ*L, intra-BLA infusion) prior to CIS exhibited a significant increase in the percentage of entries in open arms compared to the nontreated or vehicle-treated stressed group (*p* < 0.05). The stressed groups treated with vehicle (saline, p.o. or aCSF, intra-BLA infusion) showed a decrease in the percentage of time spent in open arms (Figure 1(c)). Pretreatment with Xiaoyaosan or antalarmin before CIS tended to prevent the decrease in the percentage of time spent in open arms induced by CIS. However, there were no significant differences among the groups in the percentage of time spent in open arms. We also found no significant difference in total distances travelled in 5 minutes among the groups (Figure 1(a)).

### 3.2. Effect of Xiaoyaosan on Rat Performance in the NSF Test

**Figure 2:**
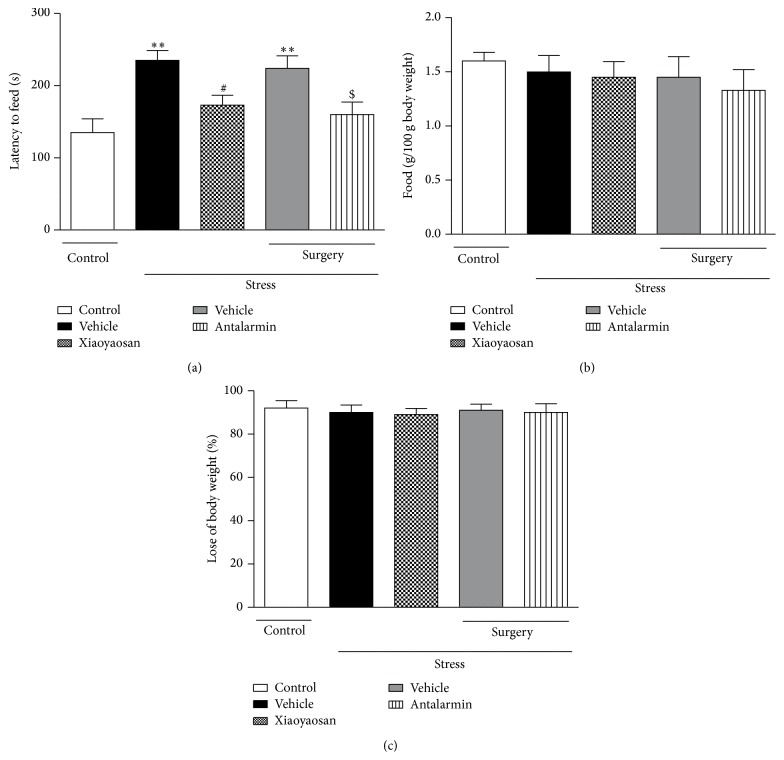
Novelty suppress feeding test. (a) Latency to begin eating. (b) Food consumption. (c) Percent loss of body weight. Values are mean ± SEM with 10 rats in each group. ^*∗∗*^ *p* < 0.01 versus the nonstressed control group. ^#^ *p* < 0.05 versus the vehicle-treated CIS group. ^$^ *p* < 0.05 versus the vehicle-treated CIS group after surgery.

To further examine the effect of Xiaoyaosan on anxiety-related behavior, we performed NSF test. After 24-h food deprivation, rats faced the conflicts between the drive to eat and the fear of the brightly lit open space, in the center of which the food pellet was placed. The latency to begin eating was used as a crucial index of anxiety-like behavior because classical anxiolytic drugs decrease it. The stressed groups treated with vehicle (saline, p.o. or aCSF, intra-BLA infusion) exhibited a significantly longer latency to begin eating than the nonstressed control group (*p* < 0.01) (Figure 2(a)). Nevertheless, pretreatment with Xiaoyaosan (3.854 g/kg, p.o.) or antalarmin (125 ng/0.5 *μ*L, intra-BLA infusion) prior to CIS reversed enhanced anxiety-like behavior as indicated by preventing the increase in latency compared to the vehicle-treated stressed group (*p* < 0.05). No differences were observed in feeding activity in the home cage or in weight loss induced by food deprivation (Figures 2(b) and 2(c)).

### 3.3. Effect of Xiaoyaosan on the Expression of CRF1R, BDNF, and pCREB in the Amygdala

**Figure 3:**
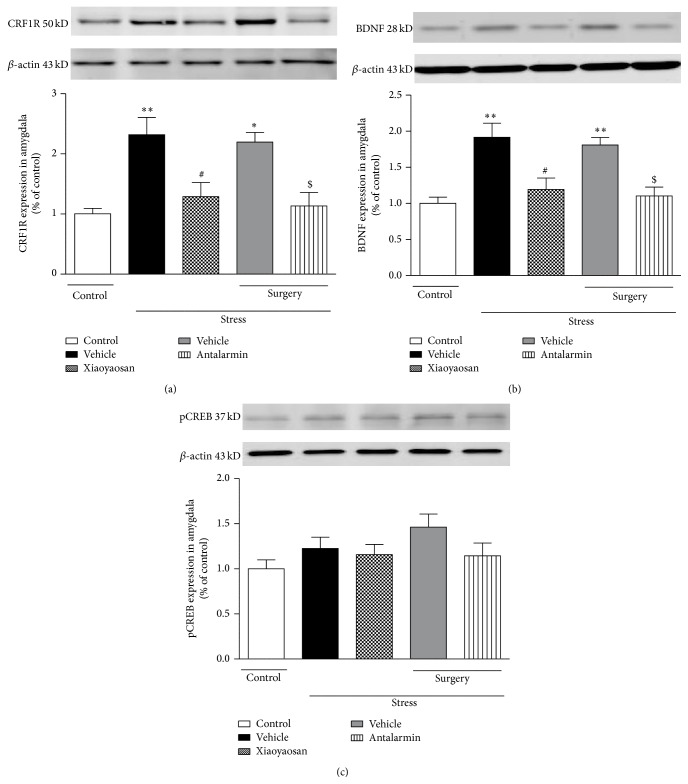
Expression of CRF1R, BDNF, and pCREB. (a) Expression of CRF1R in the amygdala. (b) Expression of BDNF in the amygdala. (c) Expression of pCREB in the amygdala. Values are mean ± SEM with 6 rats in each group. ^*∗*^ *p* < 0.05 and ^*∗∗*^ *p* < 0.01 versus the nonstressed control group. ^#^ *p* < 0.05 versus the vehicle-treated CIS group. ^$^ *p* < 0.05 versus the vehicle-treated CIS group after surgery.

In order to determine whether the effects of Xiaoyaosan on CIS-induced anxiety-like behaviors were associated with CRF1R signaling in the amygdala, which is considered to play a critical role in regulating such behaviors, we determined the expression of CRF1R, BDNF, and pCREB in different groups of rats. Compared to the nonstressed control group, rats exposed to CIS exhibited a significant increase in the expression of CRF1R in the amygdala (*p* < 0.01) (Figure 3(a)), which, however, was abated by pretreatment with Xiaoyaosan (3.854 g/kg, p.o.) or antalarmin (125 ng/0.5 *μ*L, intra-BLA infusion) (*p* < 0.05). The expression of BDNF was upregulated markedly in the amygdala in the stressed groups treated with vehicle (*p* < 0.01) (Figure 3(b)), which was reversed by pretreatment with Xiaoyaosan or antalarmin (*p* < 0.05). We found no significant difference in pCREB protein levels among the groups (Figure 3(c)).

## 4. Discussion 

Our study showed that CIS elevated anxiety-like behavior of rats as assessed in the EPM and NSF test. These rats showed a marked decrease in the percentage of entries in open arms and an increased latency to feed. Furthermore, chronic pretreatment with Xiaoyaosan or antalarmin significantly attenuated CIS-induced anxiety-like behavior. Western blotting assays additionally showed increased expression of CRF1R and BDNF in anxiety-related amygdala. Consistently, chronic pretreatment with Xiaoyaosan reversed the upregulation of CRF1R and BDNF in the amygdala of stressed rats. Based on these data, the authors conclude that the effect of Xiaoyaosan on stress-induced CRF1R hyperactivity in the amygdala may be a crucial mechanism underlying its anxiolytic-like effect.

As a potent mediator of stress- and anxiety-related behaviors, CRF1Rs are abundantly expressed in the amygdala [26]. CRF1R is a GPCR that is coupled to the stimulatory G protein Gas and can thus activate PKA and subsequently CREB [27]. However, some studies reported that both PKC [28] and PKA signaling pathways are crucial for CRF1-mediated effects in the amygdala, which may underlie anxiety-like behaviors. As novel therapeutic agents, CRF1R antagonists (CRAs) have been evaluated for depression, anxiety, and other stress-related disorders [28]. Stress can induce HPA axis activation, which could be reduced by CRAs via blocking pituitary and possibly brain CRF1Rs [29]. In our study, anxiety-like behavior was found after exposure to CIS for 14 consecutive days. This was also observed by Merali et al. [30] who demonstrated that chronic stressor exposure (daily restraint for 14 days) was associated with increased CRH and AVP expression, which were associated with anxiety and depressive symptoms. In our study, antalarmin (a CRA) reversed elevated anxiety-like behavior induced by CIS as revealed by the EPM and NSF test. This is in agreement with the study by Henry et al. [31] who demonstrated that microinfusion of the CRF1 antagonist antalarmin into the central amygdala reduced anxiogenic effect induced by immobilization stress. Moreover, a recent study showed that the administration of antalarmin into the medial amygdala exerted an anxiolytic effect in elevated T-maze test by decreasing avoidance latencies and modulating the anxiogenic effects of CRF [32]. We further examined the expression of CRF1R signaling molecules including CRF1R, pCREB, and BDNF. We found that CRF1R and BDNF were upregulated in the amygdala of rats after exposure to CIS, which are consistent with a previous report showing CIS-induced increase in BDNF expression in the BLA and a decrease in BDNF levels in area CA3 in the hippocampus [25]. Though expression of pCREB did not differ significantly in the amygdala among the groups, this might be related to the experimental design or time-dependent changes in immobilization stress. The pattern of changes in pCREB in the amygdala is complex and time dependent [33]. A previous work found that predator stress-induced increases in pCREB in the amygdala at 6 h followed by decreases at 24 h after stress [34]. Delineation of complex changes of pCREB after stress will require future examination at different time points.

On the other hand, as Xiaoyaosan contains multiple ingredients, it is possible that a different mechanism may be responsible for the anxiolytic effects of Xiaoyaosan. As a traditional Chinese remedy, Xiaoyaosan has also been studied for its chemical components. Our team has previously performed compositional analysis of Xiaoyaosan by HPLC-LTQ-Orbitrap-MS [19] and identified 8 compounds, including paeoniflorin, liquiritin, glycyrrhizic acid, ferulic acid, saikosaponins A and C, curcumin, and* Poria cocos *alcohol in Xiaoyaosan samples. The peaks of the compounds aligned with those of Xiaoyaosan extracts by using the same HPLC system, suggesting that these compounds may serve as quality control references of Xiaoyaosan [19]. Due to its multiple compounds, Xiaoyaosan targets both anxiety and depression. A previous study showed that buspirone complex with glycyrrhizic acid had a protective effect in preventing anxiety behavior [35]. Curcumin is the active component of curcuma longa and ginger and has been reported to have both antidepressant and anxiolytic effect [36–39]. A previous study has shown noticeable anxiolytic effect of curcumin against lead-induced anxiety in rats and central neuronal monoaminergic neurotransmission, especially serotonin, was highly involved [39]. Paeoniflorin, a monoterpene glycoside, was observed to exert antidepressant-like effects via inhibition of oxidative stress and Ca (2+) overload and may be of potential use in the treatment of neuropathic pain and associated insomnia [40]. Liquiritin, a flavone compound derived from* Glycyrrhiza uralensis,* has antidepressant-like activity, which may be related to defense of liquiritin against oxidative stress [41]. Recently, it has been reported that stress enhances BLA neuronal excitation, and CRF and *α*1 NE receptors could modulate such activity [42]. Moreover, many neurochemicals comprise the stress response system, including norepinephrine, serotonin, and *γ*-aminobutyric acid [43]. In addition, the methanolic extract from* Bupleurum falcatum*, derived from Xiaoyaosan, has dose-dependent antidepressant-like activity and the serotonergic and noradrenergic systems may act as the targets [44]. However, as the components are very complicated, the analysis and assessment of the disassembled prescription deserve further research. In the present study, since CRF1R and BDNF expression in the amygdala was modulated by pretreatment with Xiaoyaosan, it is possible that the anxiolytic-like effects of Xiaoyaosan are, at least in part, exerted through inhibition of the CRF1R pathway. However, the detailed mechanisms underlying the anxiolytic-like effects of Xiaoyaosan still remain to be fully elucidated, and more investigations will be required in future.

## 5. Conclusion

The results of this study show that Xiaoyaosan exerts anxiolytic-like effects in behavioral tests, which, intriguingly, may be related to CRF1R signaling in the amygdala. These findings add to our understanding of the mechanisms of the anxiolytic-like effects of Xiaoyaosan. Though our data on changes in the expression of CRF1R and BDNF in the amygdala upon treatment is preliminary, in view of previous literature on the critical role of CRF1R signaling in anxiety, we believe that the findings are valuable in guiding future studies.
